# Responsible model deployment via model-agnostic uncertainty learning

**DOI:** 10.1007/s10994-022-06248-y

**Published:** 2022-10-18

**Authors:** Preethi Lahoti, Krishna Gummadi, Gerhard Weikum

**Affiliations:** 1 Max Planck Institute for Informatics, Saarbrücken, Germany; 2 Present Address: Google Research, Zürich, Switzerland; 3 Max Planck Institute for Software Systems, Saarbrücken, Germany

**Keywords:** Trustworthy ML, Uncertainty modeling, Failure analysis

## Abstract

Reliably predicting potential failure risks of machine learning (ML) systems when deployed with production data is a crucial aspect of trustworthy AI. This paper introduces the *Risk Advisor*, a novel post-hoc *meta-learner* for estimating failure risks and predictive uncertainties of *any already-trained* black-box classification model. In addition to providing a *risk score*, the *Risk Advisor* decomposes the uncertainty estimates into aleatoric and epistemic uncertainty components, thus giving informative insights into the sources of uncertainty inducing the failures. Consequently, *Risk Advisor* can distinguish between failures caused by data variability, data shifts and model limitations and provide useful guidance on appropriate risk mitigation actions (e.g., collecting more data to counter data shift). Extensive experiments on various families of black-box classification models and on real-world and synthetic datasets covering common ML failure scenarios show that the *Risk Advisor* reliably predicts deployment-time failure risks in all the scenarios, and outperforms strong baselines.

## Introduction

### Motivation and problem

Machine learning (ML) systems have found wide adoption in mission-critical applications. Their success crucially hinges on the amount and quality of training data, and also on the assumption that the data distribution for the deployed system stays the same and is well covered by the training samples. However, this cannot be taken for granted. Saria and Subbaswamy (2019) categorize limitations and failures of ML systems into several regimes, including data shifts (between training-time and deployment-time distributions), high data variability (such as overlapping class labels) and model limitations (such as log-linear decision boundaries vs. neural ML). Trustworthy ML needs models and tools for detecting such failure risks and analyzing the underlying sources of uncertainty (Bhatt et al. 2021). Unfortunately, systems often fail silently without any warning, despite showing high confidence in their predictions (Nguyen et al. 2015; Jiang et al. 2018; Goodfellow et al. 2015).

This paper addresses the challenge of predicting, analyzing and mitigating failure risks for classifier systems. The goal is to provide the system with *uncertainty scores* for its predictions, so as to (a) reliably predict test-time inputs for which the system is likely to fail, and (b) detect the *type of uncertainty* that induces the risk, so that (c) appropriate *mitigation actions* can be pursued. Equipped with different kinds of uncertainty scores, a deployed system could improve its robustness by taking appropriate mitigation actions at deployment time, so as to smoothly handle new data points that pose difficult situations. There are three types of mitigation action that we identify and address in this paper:

(i) *Abstention* When deployment-time data points are close to the trained model’s decision boundary and there is inherent noise in the data distribution near that boundary, it would be wise to abstain from a classification and rather defer the decision to a human expert.
(ii) *More training data* When deployment-time data points fall into regions that were very sparsely populated among the training points, we face a distributional shift or “out-of-distribution” (OOD) situation. In this case, the best measure is to obtain more training points for the underpopulated regions. However, this requires understanding where the problematic regions are in the data space.
(iii) *More expressive model* Another limitation can be the learned model itself, if it has limited expressiveness in dealing with complex data distributions. Then the best measure is to replace the learner with a richer method, for example, enhancing a linear classifier with an expressive kernel or replacing it with a neural network, or increasing the number of parameters of the model within the same family.

The challenge is to determine which action is advised under which conditions. This is the problem addressed in this paper: determine the type and amount of uncertainty in deployment-time inputs, so as to decide if and which kind of mitigation is needed. We will present three types of uncertainty scores that provide guidance, each indicating the need for one of the above three actions (aleatoric uncertainty for (i), epistemic uncertainty for (ii), model uncertainty for (iii)).

**Fig. 1:**
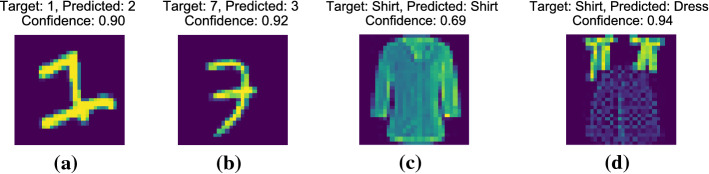
Examples of ground-truth (target) and predicted labels where (**a**, **b**) a CNN fails despite high confidence (MNIST dataset (LeCun et al. 2010)), and **c**, **d** a CNN assigns higher confidence to a misclassified sample than to a correct one (Fashion MNIST dataset Xiao et al. (2017))

### State of the art and its limitations

The standard approach for deciding whether an ML system’s predictions are trustworthy is based on confidence scores computed over predictive probabilities, such as max class probability (MCP) in neural ML (Hendrycks and Gimpel 2017) or distance from the decision boundary for SVM. However, predictive probabilities are not reliable estimates of a model’s uncertainty (Gal and Ghahramani 2016; Jiang et al. 2018; Nguyen et al. 2015; Goodfellow et al. 2015). Figure 1a, b shows two examples where a CNN model misclassifies handwritten digits (from the MNIST benchmark) while giving high scores for its (self-) confidence. Even if the confidence scores are calibrated , they may still not be trustworthy as the ordering of the confidence scores can itself be unreliable. This is because most calibration techniques (e.g., Platt et al. 1999; Guo et al. 2017), are concerned with scaling of the scores, i.e., they perform monotonic transformations with respect to prediction scores, which do not alter the ranking of confident vs. uncertain example. Figure 1c, d shows two examples where a CNN model gives higher score to a misclassified sample than to a correct one.

More importantly, confidence scores do not reflect what the model *does not know*. In Fig. 1c, d, the Fashion MNIST dataset has many positive training examples of shirts similar to (c) while hardly any examples that resemble (d)–a case where the training distribution does not sufficiently reflect the test-time data. Yet, the CNN model makes a prediction with high confidence of 0.94 (see Fig. 1d). This limitation holds even for the state-of-the-art model *Trust Score* (Jiang et al. 2018), which serves as a major baseline for this paper.

Moreover and most critically, *confidence scores* are “one-dimensional” and do not provide any insight on which type of uncertainty is the problematic issue. Thus, confidence scores from prior works are limited in their support for identifying different types of appropriate risk mitigation actions.

A common line of work for uncertainty estimation builds on Bayesian methods (Denker and LeCun 1990; Barber and Bishop 1998), or making specialized changes to the learning algorithm (e.g.,Gal and Ghahramani 2016; Depeweg et al. 2018; Lakshminarayanan et al. 2017; Shaker and Hüllermeier 2020; Malinin et al. 2021). However, these are tightly coupled to the choice of the underlying classification model and thus involve making specialized modifications to the ML pipeline. Therefore, such techniques are unsuitable for dealing with a broad variety of black-box ML systems.

### Proposed approach

**Fig. 2:**
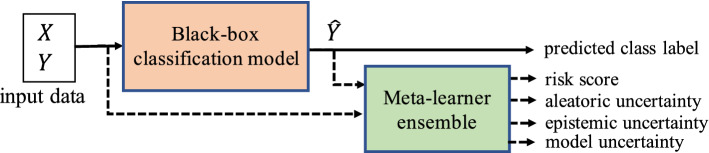
Schematic overview of the Risk Advisor framework

This paper presents *Risk Advisor*, a generic and versatile framework for reliably estimating failure risks of any already-trained black-box classification model. The *Risk Advisor* consists of a post-hoc *meta-learner* for uncertainty estimation that is separate from the underlying ML system, and can be incorporated without any code changes in the underlying ML pipeline. The *meta-learner* is model-agnostic: it can be applied to any family of black-box classifiers (e.g., deep neural networks, decision-trees, etc). Figure 2 gives a schematic overview of our framework.

In addition to providing a *risk score* that is more reliable than those of prior works, the *Risk Advisor* provides a refined analysis of the underlying types of uncertainty inducing the risks. To this end, we make use of the information-theoretic notions of *model uncertainty*, *aleatoric uncertainty* and *epistemic uncertainty* (Hora 1996; Der Kiureghian and Ditlevsen 2009; Senge et al. 2014). These concepts are fairly old, but to the best of our knowledge, have not been considered for risk analysis of black-box ML systems. Our *Risk Advisor* quantifies each of the three risk types and thus enables judicious advise on risk mitigation action, depending on the type of uncertainty inducing the risks:

- *Aleatoric uncertainty* reflects the variability of data points and the resulting noise around the classifier’s decision boundary. A high value indicates that it is inherently difficult to distinguish the output classes, and an appropriate mitigation then is to equip the deployed system with the option to *abstain* rather than forcing an output label. Figure 1a, b is a case of high aleotoric uncertainty.
- *Epistemic uncertainty* captures systematic gaps in the training samples, like regions where training samples are sparse but have a substantial population of test points after deployment. This situation can only be countered by obtaining *more training data* for the underrepresented critical regions. The unusual example of a shirt in Fig. 1d is a case of high epistemic uncertainty.
- *Model uncertainty* captures the uncertainty in model’s parameters, and is an indicator that the black-box ML system uses incorrect model class. In this situation, the proper action is to re-train the ML system with a more expressive learning model (e.g., a deep neural network instead of a log-linear model) or increase the model capacity.

The proposed *meta-learner* for estimating the different types of uncertainty in the *Risk Advisor* framework is implemented as an ensemble of *M* stochastic gradient-boosted decision trees (E-SGBT). Each stochastic gradient boosted tree (SGBT) operates on the input-output pairs of training samples and an indicator variable stating whether the trained black-box ML system misclassified the training point. The *Risk Advisor’s* analysis of uncertainty is based on the ensemble’s ability to compute aleatoric and epistemic uncertainty. All of the uncertainty scores are computed on the training data, and also at deployment time for test data alone to identify slowly evolving risks.

### Contributions

This paper’s novel contributions are as follows:

- We introduce the *Risk Advisor* framework, the first *model-agnostic* method to detect and mitigate deployment-time failure risks of *any already trained* black-box ML classifier, given access only to the base-classifier’s training data and its predictions on the training data, and coping with any kind of underlying *base-classifier* including deep neural models.
- The *Risk Advisor* is the first method that can give insights into the underlying sources of failure risks by distinguishing between ML model failures caused by distribution shifts between training data and deployment data, inherent data variability, and model limitations by leveraging the information-theoretic notions of *aleatoric* and *epistemic* uncertainty.
- Extensive experiments with synthetic and real-world datasets show that our approach successfully detects uncertainty and failure risks for many families of ML classifiers, including deep neural models, and does so better than prior baselines including the *trust score* method by Jiang et al. (2018).
- We demonstrate the *Risk Advisor*’s practical utility by three kinds of *risk mitigation* applications: (i) selectively abstaining from making predictions under uncertainty (ii) detecting out-of-distribution test-examples (iii) countering risks due to data shift by collecting more training samples in a judicious way.

A preliminary publication on this work has appeared as a short paper in the ICDM 2021 conference (Lahoti et al. 2021). This paper substantially extends our approach by performing further analysis to evaluate the *Risk Advisor*’s ability to detect the underlying uncertainties inducing the failures (see Sect. 4.3), as well as presenting how the *Risk Advisor* can effectively guide different kinds of post-deployment *risk mitigation* actions (see Sect. 5). We address three types of actions:

- *Abstaining and deferring to human:* Section 5.1 presents the applicability of uncertainty scores to selectively identify deployment-time data points on which the model should abstain.
- *Detecting out-of-distribution cases:* Section 5.2 presents the applicability of uncertainty scores to identify deployment-time data points in regions that are not sufficiently covered at training time (calling for more training data).
- *Addressing a model’s blind spots:* Section 5.3 discusses how uncertainty scores can be leveraged to guide the judicious collection of additional samples for re-training the model, with informed focus on the underrepresented regions.

## Related work

### Model-specific approaches

Most prior works addressed the task of assessing uncertainty and confidence in a model-specific manner, within the scope of a particular family of learners. These methods do not generalize to arbitrary learners and do not live up to the challenge of broad applicability.

The standard approach for predicting failure risks of ML systems is to rely on the system’s native (self-) *confidence scores*. An implicit assumption is that that most uncertain data points lie near the decision boundary, and confidence increases when moving away from the boundary. While this is reasonable to capture *aleatoric* uncertainty, this kind of confidence score fails to capture *epistemic* and *model* uncertainty (Gal and Ghahramani 2016).

A related line of work is techniques for confidence calibration such as platt scaling (Platt et al. 1999), as well as modern neural network calibration approaches such as temperature scaling (Guo et al. 2017). However, calibration approaches are concerned with rescaling the confidence scores to produce calibrated proper scores. Hence, they cannot capture model uncertainty arising due to the model’s own inductive bias. Further, like all single-dimensional notions of confidence, this is insufficient to distinguish different types of uncertainty and resulting risks. In particular, there is no awareness of epistemic uncertainty due to data shifts (Snoek et al. 2019).

Bayesian methods are a common approach to capture uncertainty in ML (Denker and LeCun 1990; Barber and Bishop 1998). Recently, a number of non-Bayesian specialized learning algorithms were proposed to approximate Bayesian methods. For instance, variational learning (Honkela and Valpola 2004; Kendall and Gal 2017), drop-out (Gal and Ghahramani 2016), and ensembles of deep neural networks (Lakshminarayanan et al. 2017). However, these models tend to be computationally expensive (by increasing network size and model parameters), and are not always practically viable. Moreover, they require specialized changes to the architecture and code of the underlying ML system.

The concepts of aleatoric and epistemic uncertainty are rooted in statistics and information theory (Hora 1996; Der Kiureghian and Ditlevsen 2009) (Hüllermeier and Waegeman (2021) is a recent overview). Senge et al. (2014) has incorporated these measures into a Bayesian classifier with fuzzy preference modeling. Shaker and Hüllermeier (2020) integrated the distinction between aleatoric and epistemic uncertainty into random-forest classifiers to enhance its robustness. Both of these works are focused on one specific ML model and do not work outside these design points, whereas Risk Advisor is model-agnostic and as such universally applicable. Shaker and Hüllermeier (2020) is included in the baselines for our experimental comparisons.

### Model-agnostic approaches

Only few works addressed the task of assessing uncertainty and deployment risks in a model-agnostic manner, dealing with all kinds of learners in a unified way. The major limitation of these works is that they provide only a single measure of uncertainty. Thus, the open challenge is to go beyond just predicting risks, by drilling down into different types of risks and providing informed guidance towards specific mitigation actions.

Several post-hoc approaches were proposed for estimating reliability scores and predicting test-time failures of already trained classifiers. Schulam and Saria (2019) proposed a post-hoc auditor to learn pointwise reliability scores. However, it is not fully model-agnostic as it relies on using gradients and the Hessian of the underlying ML model. Further, it does not differentiate between different types of uncertainty. Schelter et al. (2020) proposed a *model-agnostic* validation approach to detect *data-related* errors at serving time. However, this work focuses on errors arising from data-processing issues, such as missing values or incorrectly entered values, and relies on programmatic specification of typical data errors. Singla et al. (2021) propose visualization methods for feature extraction from robust representations to explain failures.

The closest approach to ours is *trust score* (Jiang et al. 2018), a model-agnostic method that can be applied post-hoc to any ML system. *Trust score* measures the agreement between a classifier’s predictions and the predictions of a modified nearest-neighbour classifier which accounts for density distribution. More precisely, the *trust score* for a new test-time data point is defined as the ratio between (a) the distance from the test sample to its nearest $\alpha$-high density set with a *different* class and (b) the distance from the test sample to its nearest $\alpha$-high density set with the *same* class. A major limitation of the approach, as stated in Jiang et al. (2018) and observed in that work’s experiments, is the handling of high-dimensional data starting with 1000-dimensional inputs. In these cases, the *trust score* mostly coincides with the model confidence of the underlying classifier and does not give any valued-added insight. This issue is related to the method’s approach of using point-wise distances which often degrade for high-dimensional data, and it can be tricky to choose an appropriate metric distance. Another aspect where our method improves over *trust score* is in providing different types of uncertainty scores that reveal additional insights and can guide different kinds of mitigation actions, whereas *trust score* bundles all aspects into single values.

In a broader context, classification with reject option (Bartlett and Wegkamp 2008) and selective abstention (El-Yaniv et al. 2010) are related problems, wherein classifications algorithms are extended to provide an opt-out from making a prediction in cases where the model has low confidence. However, these methods still rely on their own *confidence* scores to determine when to abstain, and thus share the limitations and pitfalls of a single-dimensional self-confidence. Similarly, the problem of detecting data shifts has been widely studied e.g., for detecting and countering covariate and label shift (Schneider et al. 2020), for anomaly detection (Ben-Gal 2010; Steinwart et al. 2005), and for algorithmic recourse (Rawal et al. 2020). These methods address data shifts, but they do not consider failure risks arising from *aleatoric* and *model* uncertainty.

## Risk advisor model

Next, we dive into the precise problem formulation and our proposed modeling approach.

### Basic concepts

#### Black-box classifier’s task

 We are given a training dataset $\mathcal {D} = \{ (x_i, y_i), \cdots ,(x_n, y_n)\} \subset \mathcal {X} \times \mathcal {Y}$, drawn from an unknown data generating distribution $\mathcal {P} \sim \mathcal {X} \times \mathcal {Y}$. The goal of the *black-box classifier* is to learn a hypothesis *h* that minimizes the empirical risk over observed training distribution $\mathcal {D}$.

1 $$\begin{aligned} h^* = \arg \min _{h} \mathbb {E}_{ (x , y) \in \mathcal {D}} \ell (h(x), y) \,, \end{aligned}$$

where $\ell (\cdot )$ is classification loss function (e.g., cross-entropy between predicted and ground-truth labels), and $\hat{y} = h(x)$ is the corresponding predicted class label.

#### Black-box classifier’s uncertainty

 The degree of uncertainty in a prediction can be measured by the Shannon entropy over the predicted class probabilities for any given test point. Higher entropy corresponds to higher uncertainty. For instance, for a binary classification task $P(y=1 \vert x)=0.5$ gives the highest entropy of $H[y \vert x]=1$.

2 $$\begin{aligned} H[Y \vert X] = - \sum _{y \in \mathcal {Y}}P(y \vert x, \mathcal {D}) \log _2 P(y \vert x, \mathcal {D}) \,. \end{aligned}$$

The overall uncertainty corresponding to the predictive task denoted as $H[Y \vert X]$ encompasses uncertainty due to aleatoric, epistemic and model uncertainty (Hora 1996; Der Kiureghian and Ditlevsen 2009; Senge et al. 2014).

### Mapping failure scenarios to uncertainties

Next, we give a brief introduction to the types of uncertainties – aleatoric, epistemic and model uncertainty–in a predictive task, and draw a connection between predictive uncertainties and common sources of failures in ML system.

**Fig. 3:**
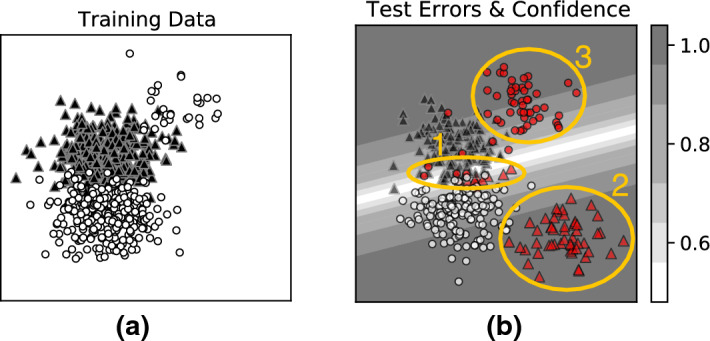
Example: (**a**) Training data for classification task (**b**) learned decision boundary of an SVM classifier and different types of test-time errors, e.g., due to (1) data variability and noise (2) data shift, and (3) model limitations

*Example:* These different kinds of uncertainty are illustrated via a synthetic example in Fig. 3. We will use this as running example to motivate the proposed approach. Consider the classification task dataset in Fig. 3. The position on x-axis and y-axis represents input features. The markers (black triangles and white circles) represent binary class labels. A linear SVM classifier, for example, would learn a decision boundary that best discriminates the two classes as shown in Fig. 3b. The test-time errors made by the model are highlighted in red. The model’s errors can be mapped to different types of uncercetainties as follows:

Firstly, in many predictive tasks *Y* can rarely be estimated deterministically from *X* due to inherent stochasticity in the dataset, a.k.a *aleatoric* uncertainty. For instance, errors arising due to inherent data variability and noise, marked as Region 1 in Fig. 3b). Such errors are inherently *irreducible* (unless additional features are collected). Additionally, there is uncertainty arising due to “lack of knowledge” about the true data generating process. For instance, consider the test errors caused by shifts in the data distribution, marked as Region 2. Such errors due to *epistemic* uncertainty can in principle be mitigated by collecting additional training data and retraining the model. Further, ML models have additional uncertainty in estimating the true model parameters given limited training data. For instance, consider *systematic* errors arising due to fitting a linear model to non-linear data, marked as Region 3. Errors due to *model* uncertainty can in principle be addressed (e.g., by training a model from a different model class).

### Design rationale

We draw inspiration from *Fano’s Inequality* (Fano 1961; Cover 1999), a classic information-theoretic inequality which when viewed from a ML perspective draws a connection between predictive uncertainty $H[Y \vert X]$, uncertainty in error prediction $H[Z \vert X]$, and probability of error $P(Z \vert X)$ of a Bayes optimal classifier, where *Z* is a random variable indicating prediction error $Z := \mathbb {I}(Y \ne \hat{Y})$.

*Fano’s* *Inequality* (Fano 1961; Cover 1999): *Consider random variables X* *and* *Y*, *where* *Y* *is related to* *X* *by the joint distribution* *P*(*x*, *y*). *Let* $\hat{Y}=h(X)$ *be an estimate of* *Y*, *with the random variable* *Z* *representing an occurrence of error, i.e.,* $Z := \mathbb {I}(Y \ne \hat{Y})$. *Fano’s inequality states that *

3 $$\begin{aligned} H[Y \vert X] \le H[Z \vert X] + P(Z \vert X) \cdot \log _2 (|\mathcal {Y}|-1) \end{aligned}$$

where $|\mathcal {Y}|$ is the number of classes, *H* is Shannon entropy, and $P(Z \vert X)$ is probability of error.

*Key Idea:* The conditional entropy $H[Z \vert X]$ and the error probability $P(Z \vert X)$ in Eq. 3 are not known, but we can approximate them by computing empirical estimates of conditional entropy $H_f[Z \vert X]$ and error probability $P_f(Z \vert X)$ of a separate *meta-learner* $f: X \rightarrow Z$ whose goal is to predict errors *Z* made by the underlying black-box classifier *h* with respect to the original classification task.

Given such a meta-learner *f*, we argue that a black-box model’s classification errors on unseen data, which relate to the uncertainty $H[Y \vert X]$, can be estimated by combining *f*’s predicted probability of error $P_f(Z \vert X)$ and *f*’s own uncertainty corresponding to predicting errors $H_f[Z \vert X]$.

**Fig. 4:**
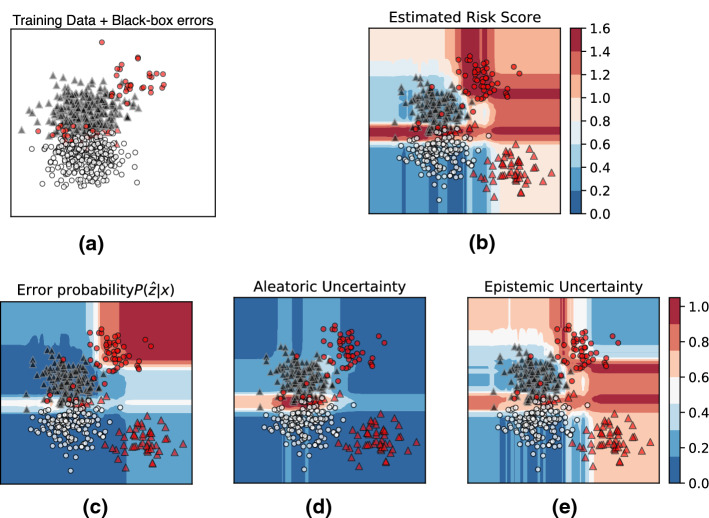
Meta-learner: **a** Training input to *meta-learner*; **b** *meta-learner’s* estimated overall *risk score* ; **c**, **d**, **e** decomposition of the overall *risk score* into its various constituting components, i.e., *model*, *aleatoric* and *epistemic* uncertainties, that capture errors due to **c** model limitations, **d** data variability and noise, and **e** data shift, respectively

*Example* Let us revisit the synthetic example of Fig. 3, looking at it from a meta-learner’s perspective. Figure 4 shows different perspectives on this setting. As earlier, red data points in each figure depict the misclassifications made by the underlying base-classifier (in this example: linear SVM). The background color in Fig. 4b–e depicts the estimated uncertainty scores of the proposed *Risk Advisor*. Regions of the input space colored in *red* depict high predicted failure risk, and regions in *blue* depict low predicted failure risk.

Figure 4a visualizes the input to the meta-learner, which consists of training datapoints *X* and the black-box model’s training errors *Z* (highlighted in red). Observe that errors due to *model limitations* (top right red points) appear as systematic errors in the input space, and are *predictable*. We argue that by training a *meta-learner* to predict black-box classification model’s errors, we can capture these systematic errors due to model limitations with meta-learner’s predicted *error probabilities* $P_f(Z \vert X)$, as shown in Fig. 4c.

Further, recall that both aleatoric and epistemic uncertainties are related to the underlying training data. We posit that the meta-learner, which is trained on the same data samples as the black-box classifier, inherits these data-induced uncertainties, and this is reflected in the meta-learner’s *aleatoric* and *epistemic* uncertainties, as shown in 4d, e.

The intuition is as follows. Consider the region near the decision boundary in Fig. 4a. As the meta-learner sees both *failure* and *success* cases of the black-box classifier in this region, the meta-learner, too, has *aleatoric uncertainty* in this region of inherent noise. Similarly, consider the test points situated far away from the training data. The meta-learner would also have significant epistemic uncertainty in its error prediction, as it has not seen any training data in this region. Thus, by estimating the meta-learner’s *own* aleatoric and epistemic uncertainty, we can indirectly capture the black-box classifier’s aleatoric and epistemic uncertainty, as shown in Fig. 4d, e, respectively. In our experiments, we will present empirical evidence of these insights.

Putting these three insights together, we propose the combined notion of *risk score*, which can capture these different types of failure risks in a unified manner, as shown in Fig. 4b. The regions with high estimated *risk score* (background color red) overlap with the regions of actual test-time errors made by the base-classifier.

In the following, Sect. 3.4 formalizes the meta-learner’s task, and presents our proposed meta-learner ensemble for the Risk Advisor. Section 3.5 discusses how to refine the overall uncertainty into informative components for different kinds of uncertainty, and compute overall *risk score*.

### Meta-learner ensemble

#### Meta-learner’s task

 Given input training samples $x \in {X}$, predicted class labels $\hat{y}:=h(x)$ of a fully trained *black-box classifier* *h*, and a random variable $Z := \mathbb {I}(Y \ne \hat{Y})$ indicating errors of the *black-box classifier* *h* with respect to the original classification task. Our goal is to learn an meta-learner $f: X \rightarrow Z$ trained to predict errors of the *black-box classifier* with respect to the original task given by

4 $$\begin{aligned} f = \arg \min _{f \in \mathcal {F}} \mathbb {E}_{ (x , z) \in \mathcal {D}} \ell (f(x), z) \end{aligned}$$

where *z* is a random variable indicating errors of the base-classifier predictor given by $z = \mathbb {I}(y \ne \hat{y})$, $\ell$ is a classification loss function. Given a newly seen test point $x^*$, the *meta-learner’s* predicted probability of error is given by $P(z \vert f, x^*)$.

However, the probability of error $P(z \vert f, x^*)$ estimated by a single meta-learner *f* can be biased due to its own uncertainty in the model parameters $P(f \vert \mathcal {D})$. Next, we show how we can obtain a reliable estimate of the black-box model’s error probability by training an ensemble of *M* independent stochastic gradient boosted trees $\mathcal {F} = \{ P(z \vert x^*, f^m )\}_{m=1}^{M}$, and computing their expectation.

#### Ensemble of Stochastic gradient boosted trees (E-SGBT)

 We consider an ensemble of *M* independent models $\mathcal {F} = \{ f^m \}_{m=1}^{M}$ such that each of the individual models $f^m$ is a stochastic gradient boosted tree (SGBT; Friedman 2002). Note that the proposed *E-SGBT* is an ensemble of ensembles, i.e., each of the *M* SGBT’s in the ensemble is itself an ensemble of *T* weak learners trained iteratively via bootstrap aggregation. To ensure minimum correlation between the *M* individual models in our ensemble, we introduce randomization in two ways. First, each of the SGBTs in the ensemble is initialized with a different random seed. Second, each of the individual SGBTs is itself an ensemble of *T* weak learners trained iteratively via bootstrap aggregation. Specifically, for each SGBT in the *E-SGBT* ensemble, at each iteration, a subsample of training data of size $\tilde{N}<N$ is drawn at random, without replacement, from the full training dataset. The fraction $\frac{\tilde{N}}{N}$ is called the sample rate. The smaller the sample rate, the higher the difference between successive iterations of the weak learners, thereby introducing randomness into the learning process.

Given *M* error probability estimates $\{P(z \vert x, f^m)\}_{m=1}^{M}$ by each of the models in the ensemble, an estimate of the probability of error $P(z \vert x , \mathcal {D})$ can be computed by taking the expectation over all the models in the ensemble:

5 $$\begin{aligned} P(z \vert x , \mathcal {D}):= \mathbb {E}_{f \in \mathcal {F}}[P(z \vert x, f, \mathcal {D})] \approx \frac{1}{M} \sum _{m=1}^{M}P(z \vert x, f^m,\mathcal {D}) \end{aligned}$$

The total uncertainty in the error prediction $H[P(z \vert x , \mathcal {D})]$ can be computed as the Shannon entropy corresponding to the estimated probability of error

6 $$\begin{aligned} H[P(z \vert x , \mathcal {D})] = - \sum _{z \in \mathcal {Z}}P(z \vert x, \mathcal {D}) \log _2 P(z \vert x, \mathcal {D}) \end{aligned}$$

### Identifying sources of uncertainty

To distinguish between different sources of uncertainty – data variariability/noise vs. data shifts between training and deployment data – we compute estimates of the *aleatoric* and *epistemic* uncertainty given an ensemble of *M* independent stochastic gradient boosted trees $\mathcal {F} = \{ f^m \}_{m=1}^{M}$. This approach was originally developed in the context of neural networks (Depeweg et al. 2018), but the idea is more general and has recently been applied to estimate uncertainties of stochastic gradient boosted trees and random forests (Malinin et al. 2021; Shaker and Hüllermeier 2020).

#### Decomposing aleatoric and epistemic uncertainty

 The main idea is that in the case of data points with epistemic uncertainty (e.g., out-of-distribution points), the *M* independent models in the ensemble given $\mathcal {F}:=\{ f^m \}_{m=1}^{M}$ are likely to yield a diverse set of predictions (i.e., different output labels) for similar inputs. In contrast, for data points with low epistemic uncertainty (e.g., in-distribution points in dense regions), they are likely to agree in their predictions. Hence, by fixing *f*, the *epistemic* uncertainty can be removed, and the *aleatoric* uncertainty can be computed by taking the expectation over all models $f \in \mathcal {F}$.

7 $$\begin{aligned} \mathbb {E}_{p(f \vert \mathcal {D})} H[P(z \vert x, f)] = \int _{\mathcal {F}} P(f \vert D) \cdot H[P(z \vert f, x)] df \end{aligned}$$

#### Aleatoric uncertainty

 Given *M* predicted probability estimates $\{P(z \vert x, f^m)\}_{m=1}^{M}$ for each of the models in the ensemble, an estimate of *aleatoric uncertainty* in Eq. 7 can be empirically approximated by averaging over individual models $f^m \in \mathcal {F}$ in our *E-SGBT* ensemble.

8 $$\begin{aligned} \mathbb {E}_{f \in \mathcal {F}} [H[P(z \vert x, f)]] \approx \frac{1}{M} \sum _{m=1}^{M} H[P(z \vert x, f^m)] \end{aligned}$$

#### Epistemic uncertainty

 Finally, *epistemic uncertainty* can be computed as the difference between *total uncertainty* and *aleatoric uncertainty*.

9 $$\begin{aligned} \underbrace{\mathcal {I}[z, f \vert x, \mathcal {D}]}_{\text {Epistemic Uncertainty}} = \underbrace{H[P(z \vert x , \mathcal {D})]}_{\text {Total Uncertainty}} - \underbrace{\mathbb {E}_{f \in \mathcal {F}} [H[P(z \vert x, f)]]}_{\text {Aleatoric Uncertainty}} \end{aligned}$$

where *total uncertainty* is the entropy corresponding to the estimated probability of error $P(z \vert x , \mathcal {D})$ given in Eq. 6.

#### Risk score

 Putting it all together, our proposed *risk score*, which captures the overall failure risks of the uncerlying black-box classifier, can be computed as the sum of (i) predicted probability of error assigned by the meta-learner, i.e., model uncertainty, (ii) epistemic uncertainty and (iii) aleatoric uncertainty.

10 $$\begin{aligned} \text {Risk Score}&:= \underbrace{P(z \vert x , \mathcal {D})}_{\text {Error probability}} + \underbrace{H[P(z \vert x)]}_{\text {Total uncertainty}} \nonumber \\&= \underbrace{P(z \vert x , \mathcal {D})}_{\text {Model Uncertainty}} + \underbrace{\mathcal {I}[z, f \vert x, \mathcal {D}]}_{\text {Epistemic uncertainty}} + \underbrace{\mathbb {E}_{f} [H[P(z \vert x, f)]}_{\text {Aleatoric uncertainty}} \end{aligned}$$

Note that this *risk score* is neither a probability nor an entropy measure, but it proves to be a very useful indicator for failure risks in our experiments. One could consider a weighted sum of each of the components to account for associated *risk costs* for each type of error. For instance, if a system designer had expert knowledge that errors due to distribution shift (i.e., epistemic uncertainty) are more harmful, she could assign more weight to the *epistemic uncertainty* component. In our experiments we assign equal weights.

#### Inference

 The meta-learner is trained on the underlying base-classifier’s training data. Given a newly seen data point $x^*$ at deployment-time, the *Risk Advisor* computes predicted error probabilities for each of the *M* models in the E-SGBT ensemble $\{P(z \vert x^*, f^m)\}_{m=1}^{M}$. These values are fed into the Risk Advisor’s estimated *error probability* in Eq. 5, *aleatoric uncertainty* in Eq. 8, *epistemic uncertainty* in Eq. 9 and *risk score* in Eq. 10. Note that at deployment-time we only expect the newly seen data point $x^*$, and the trained meta-learner.

## Experiments

In this section, we evaluate the performance of *Risk Advisor* by performing extensive experiments on real-world and synthetic datasets, and on 6 families of black-box classification models. In Subsection 4.2, we evaluate the *Risk Advisor’s* ability to *predict failure risks* at deployment time. In Subsection 4.3, we evaluate the *Risk Advisor’s* ability to *detect the sources of uncertainty* inducing the failure risk.

### Experimental setup

#### Datasets

 We evaluate the performance on the following small and large benchmark classification datasets covering a variety of common ML failure scenarios:

*High-dimensional Image Datasets:*

- *CIFAR 10:* The CIFAR-10 dataset (Krizhevsky 2009) consists of 60K color images in 10 classes, including blurred and noisy images, which are specially prone to model failures.
- *MNIST:* The MNIST dataset (LeCun et al. 2010) consists of 60K grayscale images of handwritten digits in 10 classes. Due to the variability in writing style, certain images are prone to misclassification.
- *Fashion MNIST:* The fashion MNIST dataset (Xiao et al. 2017) consists of 60K images of clothing and accessories in 10 classes, including images with rare and unusual product designs, which can be prone to errors.

*Mission-critical Fairness Datasets:*

- *Census Income:* Recent work in ML fairness has shown that models often make more errors for underrepresented groups in training data. To simulate this setting, we consider the Adult dataset (Dua and Graff 2017), a benchmark dataset in fairness literature, consisting of 49K user records. The dataset contains underrepresented groups (e.g., Female).
- *Law School:* Similarly, we use the LSAC dataset (Wightman 1998) consisting of 28K law school admission records. The classification task is to predict whether a candidate would pass the bar exam. The dataset contains underrepresented groups (e.g., “Black”).

*Distribution shift, unseen demographics/regions/domain:*

- *Census Income (Male *$\rightarrow$ *Male, Female):* To simulate distribution shift, we take the aforementioned *Census Income* dataset and exclude *female* points from the training set. Our test set consists of both Male and Female points.
- *Law School (White *$\rightarrow$ *White, Black):* Similarly, we take the aforementioned *Law School* dataset and exclude user records from the *Black* group from the training set. The test set consists of both White and Black points.
- *Heart Disease:* A common ML failure scenario is when a ML model is applied to a new geographic region. To simulate this scenario we combine four different heart disease datasets available in the UCI repository (Dua and Graff 2017) by using a subset of features overlapping between them. We use the US Cleveland heart disease dataset as our training dataset, and use it to predict heart disease on a UK statlog dataset, Hungarian (HU) and Switzerland (CH) heart disease dataset.
- *Wine Quality:* Another failure scenario is when a trained model is applied to an application domain for which it has inadequate or bad training data. To simulate this scenario, we train models on white wine, and apply it to predict quality of red wine in UCI wine dataset (Dua and Graff 2017). The classification task is to predict if the wine quality is $\ge 6$.

#### Black-box classification models

 To demonstrate the versatility of *Risk Advisor*, we evaluate it on classifiers from 6 different families, including deep neural models such as ResNet50 and CNN for the high dimensional image dataset, and classic ML algorithms such as SVM, Random Forests, Multi-layer Perceptron, and logistic regression for tabular datasets. Following are the implementation details:

- ResNet 50: The 50-layer deep residual network architecture (He et al. 2016) trained with batch size of 128 for 100 epochs.
- CNN: A convolutional neural net with 2 convolutional layers with 32, 64 hidden units, max pooling, and ReLu activations, trained with batch size 128 for 10 epochs.
- MLP: Multi-layer perceptron with 2 hidden layers with 32, 16 hidden units, batch size 64, and ReLu activations.
- SVM: support vector machines with RBF kernel and Platt scaling (Platt et al. 1999) to produce probability estimates.
- RF: a random forest with 1000 decision trees, bootstrap sampling, and max-features set to “sqrt”.
- LR: logistic regression with L2 regularization.

#### State-of-the-art baselines

 Our baseline comparison includes the underlying black-box classification model’s own (self-) *confidence scores*. Specifically, for all deep neural models, i.e., ResNET50, MLP, and CNN, we rely on the confidence score given by max class probability (DNN-MCP), as proposed by (Hendrycks and Gimpel 2017), which is a well established strong baseline. For RF’s, we rely on the *uncertainty* score, computed as per the state-of-the-art method for random forest (RF-uncertainty), as proposed by (Shaker and Hüllermeier 2020). For SVM, we rely on the standard approach of computing confidence scores over prediction probabilities from decision values after Platt scaling (SVM-Platt) (Platt et al. 1999). For LR, the confidence score is given by the distance from the decision boundary (LR-Confidence).

Our main comparison is with the state-of-the-art method *trust score* (Jiang et al. 2018). Similar to *Risk Advisor*, the *trust score* method is a model-agnostic post-hoc approach, which takes as input a black-box classifier’s predictions, and training data to produce point-wise *trust score* for newly seen test points.

While calibrating a classifier’s scores is a popular technique for producing calibrated confidence values, such techniques are rank-preserving. As all our evaluation metrics, i.e., AUROC, AUPR, and PRR (introduced later) are based on seeing different relative rankings of the scores rather than absolute values, there is no point in comparing against rank-preserving calibration techniques.

#### Implementation

 The *Risk Advisor’s* E-SGBT model is implemented as an ensemble of 10 SGBT classifiers, wherein each of the SGBT’s in the ensemble is initialized with a different random seed. For structured tabular datasets we used the Python implementation in *scikit-learn*. For high-dimensional image datasets we used the *catboost * implementation of gradient boosted decision trees (https://github.com/catboost/catboost). Best hyper-parameters are chosen via grid-search. For *Risk Advisor’s* E-SGBT model, we tune max-depth in $\{4,5,6\}$, sample-rate in $\{0.25, 0.5, 0.75\}$ and num-estimators in $\{100, 1000\}$. For *trust score*, we use the code shared by (Jiang et al. 2018) and perform grid search over the parameter space reported in the paper. Each dataset is separated into 70:30 stratified training and test splits. All numerical features in the datasets are standardized to unit variance and and all categorical features are transformed via one-hot encoding. On the training split, we perform 5-fold cross-validation to find the best hyper-parameters via grid search. Once hyper-parameters are tuned, we refit the model using best hyper-parameters and report results on the independent test set.

**Table 1:** AUROC for predicting test-time failure risks: values in the table are area under ROC curve (AUROC)

Dataset	CIFAR 10	Fashion	MNIST	Census income				Law school				Wine quality				Heart disease				Census income				Law school			
		MNIST										White → White, red				US → US, UK, CH, HU				Male → Male, Female				White → White, Black			
Black-box (BBox)	ResNet50	CNN	CNN	LR	MLP	RF	SVM	LR	MLP	RF	SVM	LR	MLP	RF	SVM	LR	MLP	RF	SVM	LR	MLP	RF	SVM	LR	MLP	RF	SVM
Classification model																											
LR-Confidence	–	–	–	**0.80**	–	–	–	0.70	–	–	–	0.62	–	–	–	0.70	–	–	–	0.78	–	–	–	0.71	–	–	–
SVM-Platt Platt et al. (1999)	–	–	–	–	–	–	0.83	–	–	–	0.75	–	–	–	0.72	–	–	–	0.76	–	–	–	0.85	–	–	–	0.75
DNN-MCP Hendrycks and Gimpel (2017)	0.78	0.90	**0.98**	–	**0.80**	–	–	–	0.76	–	–	–	0.57	–	–	–	0.68	–	–	–	0.78	–	–	–	0.76	–	–
RF-uncertainty Shaker and Hüllermeier (2020)	–	–	–	–	–	0.75	–	–	–	0.71	–	–	–	0.65	–	–	–	**0.75**	–	–	–	0.69	–	–	–	0.70	–
Trust score Jiang et al. (2018)	0.64	0.88	0.96	0.65	0.65	0.71	0.71	0.69	0.69	0.83	0.81	**0.67**	0.71	0.69	0.73	0.70	0.66	0.65	0.69	0.62	0.62	0.67	0.67	0.70	0.68	0.83	0.82
Risk score (Proposed)	**0.80**	**0.92**	**0.98**	**0.80**	**0.80**	**0.87**	**0.86**	**0.83**	**0.77**	**0.86**	**0.86**	0.66	**0.75**	**0.74**	**0.75**	**0.78**	**0.72**	0.72	**0.79**	**0.79**	**0.79**	**0.87**	**0.87**	**0.83**	**0.77**	**0.86**	**0.85**

Best results are highlighted in bold

Higher values are better

**Table 2:** AUPR for Predicting test-time failure risks: values reported in the table are area under PR curve (AUPR)

Dataset	CIFAR 10	Fashion	MNIST	Census income				Law school				Wine quality				Heart disease				Census income				Law school			
		MNIST										White → White, red				US → US, UK, CH, HU				Male → Male, Female				White → White, Black			
Black-box (BBox)	ResNet50	CNN	CNN	LR	MLP	RF	SVM	LR	MLP	RF	SVM	LR	MLP	RF	SVM	LR	MLP	RF	SVM	LR	MLP	RF	SVM	LR	MLP	RF	SVM
Classification model																											
Random Baseline	0.30	0.11	0.01	0.16	0.16	0.22	0.22	0.19	0.15	0.28	0.31	0.31	0.37	0.35	0.37	0.28	0.20	0.19	0.25	0.18	0.19	0.26	0.28	0.21	0.17	0.31	0.34
LR confidence	–	–	–	0.39	–	–	–	0.37	–	–	–	0.42	–	–	–	0.42	–	–	–	0.40	–	–	–	0.41	–	–	–
SVM-Platt Platt et al. (1999)	–	–	–	–	–	–	0.52	–	–	–	0.48	–	–	–	0.56	–	–	–	0.47	–	–	–	0.63	–	–	–	0.50
DNN-MCP Hendrycks and Gimpel (2017)	**0.58**	0.46	0.31	–	**0.38**	–	–	–	0.38	–	–	–	0.43	–	–	–	0.39	–	–	–	0.39	–	–	–	0.40	–	–
RF uncertainty Shaker and Hüllermeier (2020)	–	–	–	–	–	0.42	–	–	–	0.43	–	–	–	0.46	–	–	–	0.40	–	–	–	0.42	–	–	–	0.44	–
Trust score Jiang et al. (2018)	0.43	0.47	**0.36**	0.22	0.21	0.33	0.33	0.37	0.29	0.66	0.64	**0.49**	0.54	0.51	**0.59**	0.47	**0.39**	0.33	0.40	0.23	0.23	0.35	0.37	0.41	0.30	**0.66**	**0.67**
Riskscore (Proposed)	**0.59**	**0.52**	**0.36**	**0.40**	0.37	**0.61**	**0.60**	**0.56**	**0.44**	**0.67**	**0.66**	0.45	**0.61**	**0.56**	0.58	**0.54**	0.37	**0.45**	**0.58**	**0.42**	**0.42**	**0.68**	**0.69**	**0.58**	**0.42**	**0.66**	0.66

Best results are highlighted in bold

Higher values are better

### Predicting test-time failure risks

In this experiment, we evaluate the ability of the *Risk Advisor* to successfully flag the test points that are likely to be misclassified by the underlying ML classfication system.

#### Metrics

 As we are interested in predicting failure risks, the performance on this task can be assessed via the Risk Advisor’s ability to detect *suspicious* or *untrustworthy* (Jiang et al. 2018)] examples, i.e., the test examples that are incorrectly classified by the base-classifier. We can formulate this task as a binary classification task, and measure the quality of failure prediction using standard metrics used in the literature (Hendrycks and Gimpel 2017): area under ROC curve (AUROC) and area under precision recall curve (AUPR), where the base-classifier’s misclassifications are chosen as the positive class. We choose AUROC as it has intuitive interpretation: the probability that a misclassified example has a higher *risk score* value than a correctly classified example. Additionally, we choose AUPR for the evaluation as it is more informative than AUROC in the scenarios where the number of examples in the positive class (i.e., misclassified examples) is very small. The results on AUPR are related to the metric on detecting suspicious examples by Jiang et al. (2018), where the authors plot precision for varying percentiles of (negative) *trust score*. In contrast, the PR curve plots precision for varying thresholds of recall, and can be summarized by AUPR which measures average precision over various thresholds of recall between 0 and 1.

#### Results

 Tables 1 and 2 shows a comparison between the black-box models’ own *confidence scores* (Hendrycks and Gimpel 2017; Platt et al. 1999; Shaker and Hüllermeier 2020), *trust score* (Jiang et al. 2018) and the *Risk Advisor*’s estimated *risk score*, for all combinations of datasets and black-box models. Table 1 reports AUROC for detecting test-set errors of the underlying black-box classifiers. Best values are marked in bold. We make the following observations.

First, we observe that AUROC values for all the methods are higher than a random baseline (AUROC of 0.5), indicating that all the approaches are informative in detecting test errors. Second, the proposed *risk score* consistently *outperforms* black-box models’ own confidence scores (barring a few exceptions). This holds true for all families of black-box classifiers including deep neural models and Random Forests, which build on DNN-MCP (Hendrycks and Gimpel 2017) and RF-uncertainty (Shaker and Hüllermeier 2020). Finally, we observe that our Risk Advisor’s *risk scores* consistently *outperform trust scores* by a significant margin, for all the datasets and all families of black-box classifiers. Similar trends hold for the AUPR metric as shown in Table 2.

**Fig. 5:**
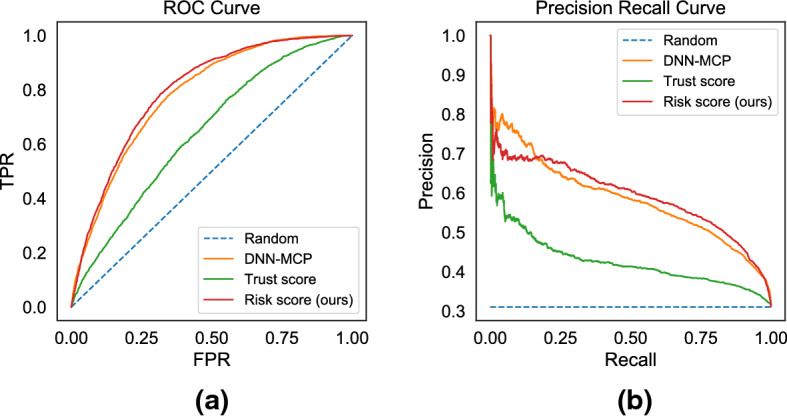
Predicting Failure Risks: Comparison of **a** ROC curves and **b** Precision Recall curves on CIFAR 10 dataset for base-classifier ResNet50

**Fig. 6:**
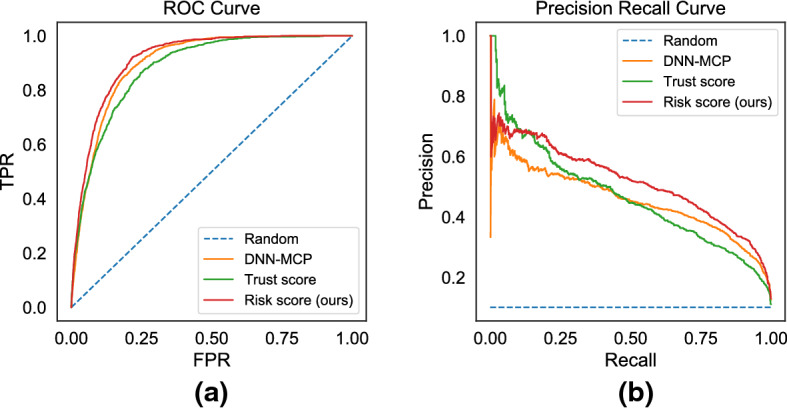
Predicting Failure Risks: Comparison of **a** ROC curves and **b** Precision Recall curves on Fashion MNIST dataset for base-classifier CNN

Figures 5 and 6 show the ROC curve and the Precision-Recall curve on the two largest datasets for neural base-classifiers: CIFAR 10 with base-classifer ResNet50 and Fashion MNIST with base-classifier CNN. Consistent with the summary AUROC and AUPR metrics in Tables 1 and 2, we observe that all methods are better than a random baseline. The proposed *risk score* is the most informative, followed by the base-classifiers’ native *confidence scores*(DNN-MCP) and *trust score* being last.

### Detecting sources of uncertainty

In this experiment, we evaluate how well the *Risk Advisor* can successfully detect the underlying sources of uncertainty inducing the failure risk. However, for real-world datasets and complex black-box models it is difficult to collect ground truth (for evaluation) on which errors are due to inherent data complexity or model limitations. Hence, in this section we only focus on systematically generated synthetic data.

To this end, we generate synthetic datasets covering a variety of ML failure scenarios including errors due (i) distribution shift between training and test data (ii) inherent data variability and reflecting noise in class labels (iii) black-box classifier’s model limitations (e.g., applying a linear model to non-linear decision boundary). We then evaluate if the *Risk Advisor’s* estimates for *epistemic*, *aleatoric* and *model* uncertainty can correctly capture the corresponding test-time errors made by the black-box classification model.

#### Errors due to distribution shift

**Fig. 7:**
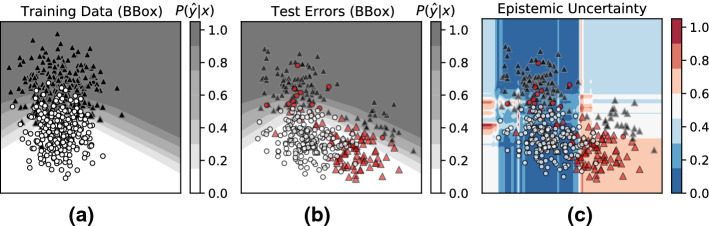
Errors from distribution shift: Risk-Advisor’s epistemic uncertainty correctly identifies test points far away from training distribution

 In order to simulate a distribution shift scenario, we draw points from a mixture of two Gaussians. For the training points we set the mixture coefficient for one of the Gaussians to zero; for the test points both mixture components are active. This way, we are able to construct a dataset containing out-of-distribution test points as shown in Fig. 7. Figure 7a visualizes the training data and the decision boundary learned by a 2-layer feed-forward neural network (NN). Figure 7b visualizes the test data. Test errors of the NN are highlighted in red. Observe that the NN *misclassifies* out-of-distribution test points while (incorrectly) reporting high confidence. The contour plot in Fig. 7c visualizes *Risk Advisor’s* estimated *epistemic* uncertainty.

Ideally, we would like to see that the *epistemic* uncertainty increases as we move towards the sparse regions of the training data, and that it is high for out-of-distribution regions. Despite some noise, we clearly see this trend: regions of low epistemic uncertainty (i.e., dark-blue regions) coincide with the dense in-distribution test points. *Epistemic* uncertainty increases as we move towards sparse regions, and the values are especially high for out-of-distribution regions. This can be seen in the right half of Fig. 7c where the estimated epistemic uncertainty is between 0.4 and 0.6 in comparison to the entire left half of the heatmap, which has estimated uncertainty between 0.0 and 0.2. The estimated scores are not perfect, as there are little to no training points in the upper and lower right regions, but the epistemic uncertainty values are useful for identifying out-of-distribution regions.

#### Errors due to data variability and noise

**Fig. 8:**
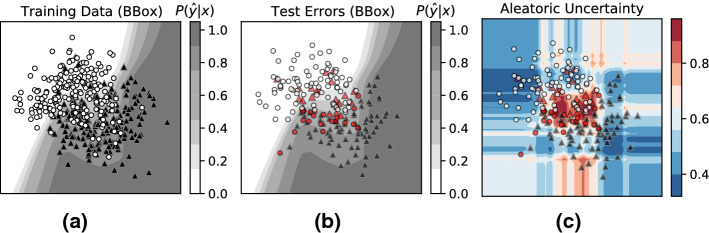
Errors from data variability and noise: Risk Advisor’s aleatoric uncertainty correctly identifies test points in the regions with class overlap

 To simulate a dataset with inherent noise, we draw points from the classic two-moons dataset (Pedregosa et al. 2011), and add Gaussian noise with standard deviation 0.5 to the dataset as shown in Fig. 8. Figure 8a visualizes the training data and the decision boundary learned by a 2-layer feed-forward neural network (NN). Figure 8b visualizes the test data. Test-errors are highlighted in red.

The contour plot in Fig. 8c visualizes estimated *aleatoric* uncertainty. Ideally, we would expect that *aleatoric* uncertainty is high for the regions with data variability i.e., regions with large class overlap. We clearly see this trend: the estimated *aleatoric* uncertainty is high for the test points near the decision boundary, with high class overlap. The regions of high aleatoric uncertainty (i.e., dark-red regions) coincide with the test-errors due to data-variability and noise.

#### Errors due to black-box classifier’s model limitations

**Fig. 9:**
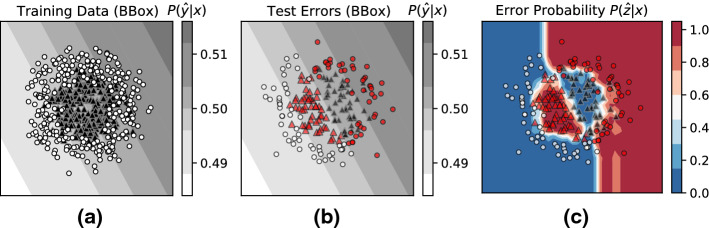
Errors from model limitations: *Risk Advisor’s* estimated error probability $P(z \vert x)$ correctly identifies errors due to model limitation

 In order to simulate this scenario, we construct a classification dataset with a non-linear decision boundary, i.e., two concentric circles (Pedregosa et al. 2011). We then fit a misspecified classification model to the task, i.e., a logistic regression classifier with a (log-)linear decision boundary as shown in Fig. 9. The contour plot in Fig. 9a visualizes the training data and the learned decision boundary. Figure 9b visualizes the test data. Test-set errors made by the black-box model are highlighted in red.

The contour plot in Fig. 9c visualizes the *Risk Advisor’s* predicted *Error probability* ($P(\hat{z} \vert x)$). Ideally, we would expect the *Risk Advisor* to assign a higher error score for regions of the input space where the black-box classifier makes errors due to its model limitations. We clearly see this trend: the *Risk Advisor* correctly identifies the regions where the black-box classifier is likely to make *systemayic errors* due to its incorrect model class, i.e., linear decision boundary. This is especially remarkable given that the *Risk Advisor* has no knowledge of the model family of the underlying black-box model (e.g., whether it is log-linear model or a neural network). In spite of having no information about the underlying model (other than its predictions), the *Risk Advisor* is able to correctly capture the *model uncertainty*.

### Efficiency and Scalability

This section discusses the efficiency and scalability of the proposed *Risk Advisor* model. Recall that the learning of the *Risk Advisor* is by an ensemble of M independent stochastic gradient boosted classifiers (SGBT), each operating on a randomly sampled subset of data points. The M classifiers can be trained in parallel. Each SGBT training, on the other hand, is a sequential computation. However, the training time can be tuned by controlling the number of training iterations, the depth of the decision trees, and the sampling rate.

All our experiments were performed with an ensemble of 10 SGBT’s, each trained for 100 or 1000 iterations, maximum depth of 4, 5 or 6, and a sampling rate of 0.25, 0.5 or 0.75. For structured tabular datasets we used the Python implementation in *scikit-learn*. For high-dimensional image datasets we used the *catboost * implementation of gradient boosted decision trees (https://github.com/catboost/catboost).

Empirically, the training time for the *risk score* estimator was one or two orders of magnitude lower than the training of the underlying base-classifier. For the largest dataset (CIFAR 10), the training time of the base-classifier (ResNet50) was a couple of hours, whereas the training of the *Risk Advisor* took only a few minutes. For the largest tabular dataset (Census Income), the training time of the base-classifier was in the order of minutes, whereas the training of the *Risk Advisor* was in the order of seconds.

## Applications to risk mitigation

Next, we investigate the *Risk Advisor’s* applicability to a variety of applications for *risk mitigation*, including (i) selectively abstaining under uncertainty (Subsection 5.1) (ii) detecting out-of-distribution test examples (Subsection 5.2) and (iii) mitigating risk by judiciously collecting additional samples for re-training the system (Subsection 5.3).

### Risk mitigation by selective abstention

A benefit of predicting failure risks at deployment time is that we can take meaningful *risk mitigation* actions. For instance, if we expect that a ML system is likely to misclassify certain deployment/test-points, we can ask the ML system to abstain from making predictions and instead forward these data points to a fall-back system or human expert. In this experiment, we simulate the latter scenario as follows.

#### Setup and metric

 We generate a ranking of all the test points by ordering them according to the scores assigned by each approach, i.e., black-box model’s *confidence score* (ascending order), *trust score* (ascending order), and *Risk Advisor*’s *risk score* (descending order), respectively. We then use these rankings to choose test points to defer to an *oracle*, in which case the ML systems predictions are replaced with the *oracle’s* labels. This setup allows us to compute an *Accuracy-Rejection curve* (AR curve) (Malinin 2019; Bartlett and Wegkamp 2008; El-Yaniv et al. 2010). An oracle would rank all *misclassification* points first, thus leading to an optimal curve (Oracle). A random ranking would yield a linearly decreasing AR curve from *base-error-rate* to 0.0 (Random). An AR curve produced by informative estimates of failure risks would lie between the *Random* and *Oracle* curves.

**Fig. 10:**
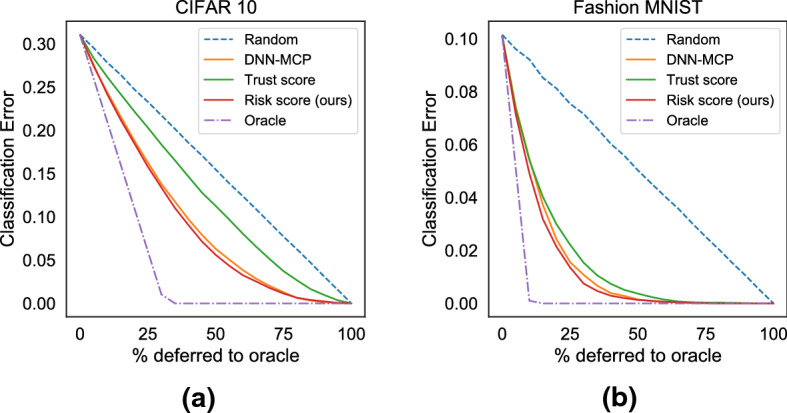
Selective Abstention: Comparison of Accuracy-Rejection curves on **a** CIFAR 10 dataset for base-classifier ResNet50 and **b** Fashion MNIST dataset for base-classifier CNN

The AR curve can be summarized with a metric *prediction rejection ratio* (PRR) (Malinin 2019). It measures the degree to which the uncertainty scores are informative by the ratio of the area between the *uncertainty estimator* and *random* curves, to the area between *oracle* and *random* curves. The *PRR* value lies between 0.0 and 1.0, where 1.0 indicates perfect ordering and 0.0 indicates uniformly random ordering.

$$\begin{aligned} \text {PRR} = \frac{\text {AU}_\text {uncertainty} - \text {AU}_\text {random}}{\text {AU}_\text {oracle} - \text {AU}_\text {random}} \end{aligned}$$

#### Results

 Table 3 shows a comparison between the black-box models’ own *confidence score* (Hendrycks and Gimpel 2017; Platt et al. 1999; Shaker and Hüllermeier 2020), *trust score* (Jiang et al. 2018) and the proposed *risk scores*. Values in the table are PRR values for all combinations of datasets and models. Best results are highlighted in bold. We make the following observations:

First, all methods under comparison have a PRR $>0$, indicating that all the approaches are informative and better than a random baseline (with random abstention). Second, *risk scores* consistently yield the *best PRR* across all datasets and classification models (barring a few exceptions). There is no clear winner between *trust score* and each of the black-box classifiers’ native *confidence score*.

Figure 10 shows the Accuracy-Rejection (AR) curves for the largest two datasets: CIFAR 10 and Fashion MNIST with base-classifiers ResNet50 and CNN, respectively. The values on the x-axis are the percentage of datapoints deferred to the oracle, and the y-axis shows the error rate of the base-classifier. Consistently with our observations on the PRR metric in Table 3, we observe that all approaches are better than a random baseline (i.e., with random abstention). The proposed *risk score* is the most informative, followed by the base-classifiers’ native confidence scores and *trust score* being last.

**Table 3:** Risk mitigation by selective abstention. values in the table are prediction rejection ratio (PRR)

Dataset	CIFAR 10	Fashion	MNIST	Census income				Law school				Wine quality				Heart disease				Census income				Law school			
		MNIST										White → White, red				US → US, UK, CH, HU				Male → Male, Female				White → White, Black			
Black-box (BBox) Classification model	ResNet50	CNN	CNN	LR	MLP	RF	SVM	LR	MLP	RF	SVM	LR	MLP	RF	SVM	LR	MLP	RF	SVM	LR	MLP	RF	SVM	LR	MLP	RF	SVM
LR-Confidence	–	–	–	0.59	–	–	–	0.39	–	–	–	0.23	–	–	–	0.41	–	–	–	0.56	–	–	–	0.41	–	–	–
SVM-Platt Platt et al. (1999)	–	–	–	–	–	–	0.66	–	–	–	0.51	–	–	–	0.44	–	–	–	0.52	–	–	–	0.69	–	–	–	0.50
DNN-MCP Hendrycks and Gimpel (2017)	0.57	0.80	**0.96**	–	**0.60**	–	–	–	0.52	–	–	–	0.14	–	–	–	0.36	–	–	–	0.56	–	–	–	0.53	–	–
RF-uncertainty Shaker and Hüllermeier (2020)	–	–	–	–	–	0.51	–	–	–	0.41	–	–	–	0.30	–	–	–	**0.50**	–	–	–	0.38	–	–	–	0.41	–
Trust score Jiang et al. (2018)	0.29	0.77	0.91	0.31	0.30	0.41	0.42	0.38	0.38	0.67	0.63	**0.34**	0.41	0.38	0.46	0.39	0.32	0.30	0.38	0.24	0.25	0.34	0.35	0.41	0.36	0.66	0.63
Risk score (Proposed)	**0.59**	**0.83**	**0.96**	**0.61**	**0.60**	**0.74**	**0.73**	**0.65**	**0.54**	**0.73**	**0.71**	0.32	**0.50**	**0.47**	**0.50**	**0.57**	**0.45**	0.44	**0.57**	**0.57**	**0.58**	**0.74**	**0.74**	**0.66**	**0.53**	**0.73**	**0.70**

Best results are highlighted in bold

Higher values are better

**Table 4:** Detecting out-of-distribution (OOD) test examples: values in the table are AUROC for OOD detection

Dataset	Wine quality				Heart disease				Census income				Law school			
	White → White, red				US → US, UK, CH, HU				Male → Male, Female				White → White, Black			
BBox classification model	LR	MLP	RF	SVM	LR	MLP	RF	SVM	LR	MLP	RF	SVM	LR	MLP	RF	SVM
LR-Confidence	0.33	–	–	–	0.66	–	–	–	0.45	–	–	–	0.68	–	–	–
SVM-Platt Platt et al. (1999)	–	–	–	0.81	–	–	–	0.67	–	–	–	0.42	–	–	–	0.66
MCP Hendrycks and Gimpel (2017)	–	0.25	–	–	–	0.63	–	–	–	0.42	–	–	–	0.61	–	–
RF-epistemic uncertainty Shaker and Hüllermeier (2020)	–	–	**0.84**	–	–	–	0.55	–	–	–	**0.64**	–	–	–	0.62	–
Trust score Jiang et al. (2018)	0.61	0.65	0.62	0.64	0.66	**0.65**	0.62	0.64	0.42	0.42	0.42	0.42	0.66	0.68	0.67	0.62
Epistemic uncertainty	**0.81**	**0.87**	0.82	**0.91**	**0.67**	0.54	**0.74**	**0.72**	**0.51**	**0.57**	0.54	**0.48**	**0.72**	**0.70**	**0.72**	**0.68**

Best results are highlighted in bold

Higher values are better. The notation $A \rightarrow A, B, C \ldots$ means that the training data had only points with attribute value *A* but the test set had points with values $A, B, C \ldots$

### Detecting data shift at deployment time

In this experiment, we evaluate how well the *Risk Advisor’s* estimate of *epistemic uncertainty* can be used can successfully detect data shift at deployment time, i.e., detect test points coming from a different distribution than the one which the model was trained on.

#### Setup and metric

 To this end, we narrow our focus on the four datasets on out-of-distribution (OOD) test points for which we have ground truth labels shown in Table 4. Given a combined test dataset consisting of both in-distribution and out-of-distribution test points, the question at hand is to what extent the *Risk Advisor*’s estimated *epistemic uncertainty* can effectively separate in-distribution and out-of-distribution test points. As we have ground truth for out-of-distribution test points and we have ensured that there are equal numbers of in/out distribution test points, we can use the area-under-the-ROC-curve metric (AUROC) to evaluate the ability of each of the scores to separate OOD points from in-distribution points. Intuitively, AUROC measures the degree to which each of the confidence scores ranks a randomly chosen OOD data point higher than a randomly chosen non-OOD point.

#### Results

 Table 4 shows a comparison between the black-box model’s own *confidence* score, *trust score*, and the *Risk Advisor*’s estimated *epistemic uncertainty*. Unlike baseline methods for DNN, SVM, and LR, the baseline for computing uncertainty of RF by (Shaker and Hüllermeier 2020) can decompose the overall uncertainty into aleatoric and epistemic components. Thus, for RF, we rely on the *epistemic* uncertainty estimates. We make the following observations.

First, observe that *epistemic uncertainty* consistently outperforms both the black-box model’s own *confidence score* and *trust score* across all datasets and classification methods, with a significant margin. Further *Risk Advisor’s* epistemic uncertainty is competitive with RF-epistemic uncertainty, which is model-specific and has full access to the RF classifier. This supports our argument that a post-hoc *meta-learner* trained to compute uncertainties, is a viable alternative to replacing the underlying black-box ML classifier, which may not be feasible in production practice. Second, observe that for the Wine and Census Income datasets, the DNN-MCP (Hendrycks and Gimpel 2017) and LR *confidence score* has AUROC substantially lower than 0.5, that is, significantly worse than the random baseline, implying that black-box models incorrectly assign higher confidence scores for OOD points than for in-distribution points. In contrast, the AUROC values for the *epistemic uncertainty* is significantly above 0.5 in most cases. An exception is the Census Income dataset where it is only slightly above 0.5 and still performs much better than all baselines. Our hypothesis is that this dataset poses difficulties by its sophisticated feature distribution, which makes it harder to identify that the withheld female population represents an OOD case.

Overall, this shows that the *Risk Advisor* mostly assigns higher *epistemic* uncertainty for OOD test points than for in-distribution test points. This is an important property, as it indicates that a ranked ordering of test points by *epistemic uncertainty* can be used in a deployed application to detect out-of-distribution test points (given an application-specific threshold). For these critical data points, the system could resort to a human expert (or other fall-back option), and thus enhance trustworthiness of the ML system.

### Risk mitigation by judicious sampling and retraining

Being able to identify black-box classifier’s epistemic uncertainty enables another type of mitigation action: to mitigate risks due to evolving data by judiciously collecting more training examples and re-training the ML system.

We acknowledge the large body of literature on active sampling and domain adaptation in this context. In our experiment the goal is not to compare with these existing techniques, but rather to demonstrate an application of the *Risk Advisor*’s epistemic uncertainty, which can be achieved without making any changes to the underlying black-box classification system.
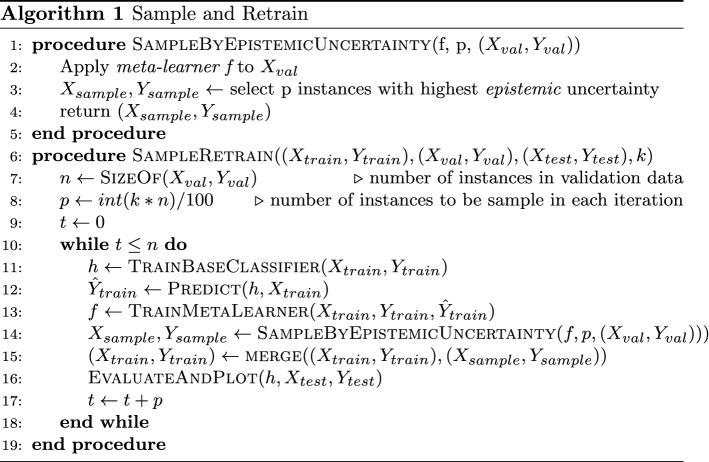

**Fig. 11:**
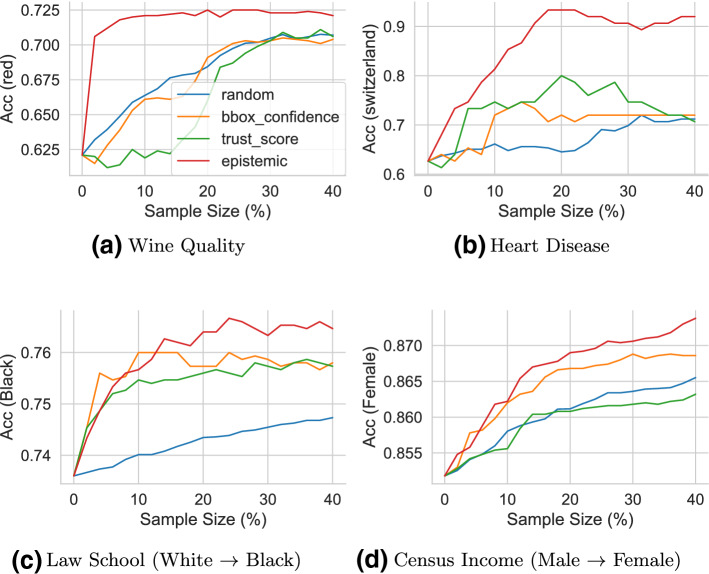
Addressing distribution shift: Comparison of various sampling strategies to selectively sample data points and retrain the black-box classification model. Curves that grow higher and faster from left to right are better

#### Setup and metric

 In this experiment, we fix the black-box classifier to logistic regression, and we assume that we have access to an untouched held-out set of labeled samples (different from training and test set). Our goal is to evaluate if the performance of the underlying black-box classifier can be improved for out-of-distribution test points by additional sampling and re-training the ML system on (a subset of) these held-out points. To evaluate the performance, we use the black-box classifier’s improvement in accuracy for out-of-distribution test points.

We compare different sampling strategies by selecting data points from the with-held set in different orders based on three criteria: the *LR-Confidence*, *trust score*, and the *Risk Advisor*’s *epistemic uncertainty*. For each approach, we first compute point-wise scores for all the points in the held-out set (different from training and test set, kept aside for sampling experiment). We then order the points in the held-out set according to these scores, i.e., *LR-confidence* (ascending order), *trust score* (ascending order), and *Risk Advisor*’s *epistemic uncertainty* (descending order), respectively. Next, at each round of an iterative sampling, we select $k\%$ points from the held-out set (with replacement), and re-train the ML system. Algorithm 1 shows pseudo-code for the iterative sampling and retraining procedure.

#### Results

 Figure 11 shows results averaged over 5 independent runs. The x-axis shows the percentage of additional points sampled from the held-out set for re-training, and the y-axis shows the corresponding improved accuracy for the OOD group (e.g., accuracy on red wine for the Wine dataset). Ideally, we would expect the accuracy to rise higher with as few additional training points as possible. We make the following observations.

First, as we sample and retrain on additional points from the held-out data, the accuracy for OOD test-points increases for all the approaches on all datasets. However, the percentage of additional samples required to achieve similar performance differs across approaches. Not surprisingly, *random* sampling is the slowest improving approach for 3 out of 4 datasets, followed by *trust score* and *confidence score*. The Risk Advisor’s sampling by *epistemic* uncertainty consistently outperforms on all datasets, by a large margin. For instance, on the Heart Disease dataset *epistemic* uncertainty achieves 30 percentage points (pp) improvement in accuracy (from 0.6 to 0.9) for an additional 20% samples from the held-out set. In contrast, all the other approaches stagnate around 0.7 even for an additional 40% samples. Similarly, on the Wine Quality dataset we see an improvement of 10 pp for an additional 10% samples, while other approaches do not reach this improvement even for additional 40% of samples. We observe similar trends across approaches for Law School and Census Income datasets, albeit with smaller gains.

## Conclusion

This paper presented the *Risk Advisor* model for detecting and analyzing sources of uncertainty and failure risks when a trained classifier is deployed for production usage. The Risk Advisor treats the base-classifier as a black-box model, and this model-agnostic approach makes it a highly versatile and easy-to-deploy tool. In contrast to the prior state-of-the-art (including the main baseline trust score (Jiang et al. 2018)), the Risk Advisor goes beyond providing a single measure of uncertainty, by computing refined scores that indicate failure risks due to data variability and noise, data shifts between training and deployment, and model limitations. Extensive experiments on various families of black-box classifiers and on real-world datasets covering common ML failure scenarios show that the Risk Advisor reliably predicts deployment-time failure risks in all the scenarios, and outperforms strong baselines. Thereby, we believe the Risk advisor, with its ability to audit and identify potential regions of failure risks would be a useful asset for the responsible ML toolbox.

## Data Availability

All the datasets used in this paper are publicly available in open data repositories at https://archive.ics.uci.edu/ml/datasets.php (Census Income, Wine Quality and Heart Disease datasets), http://yann.lecun.com/exdb/mnist/ (MNIST digits dataset), https://www.cs.toronto.edu/~kriz/cifar.html (CIFAR 10) dataset, https://github.com/zalandoresearch/fashion-mnist (Fashion MNIST dataset) and http://www.seaphe.org/databases.php (LSAC Law School dataset).

## Code availability

All the code used in this paper is available at https://github.com/plahoti-lgtm/RiskAdvisor.
